# Cardio-Rheumatology Insights Into Hypertension: Intersection of Inflammation, Arteries, and Heart

**DOI:** 10.1093/ajh/hpae098

**Published:** 2024-07-26

**Authors:** Shadi Akhtari, Paula J Harvey, Lihi Eder

**Affiliations:** Division of Cardiology, Department of Medicine, Women’s College Hospital, Toronto, ON, Canada; Women’s College Research Institute, Women’s College Hospital, Toronto, ON, Canada; Department of Medicine, University of Toronto, Toronto, ON, Canada; Division of Cardiology, Department of Medicine, Women’s College Hospital, Toronto, ON, Canada; Women’s College Research Institute, Women’s College Hospital, Toronto, ON, Canada; Department of Medicine, University of Toronto, Toronto, ON, Canada; Division of Rheumatology, Department of Medicine, Women’s College Hospital, Toronto, ON, Canada; Women’s College Research Institute, Women’s College Hospital, Toronto, ON, Canada; Department of Medicine, University of Toronto, Toronto, ON, Canada

**Keywords:** atherosclerotic cardiovascular risk, cardio-rheumatology, hypertension, inflammation, inflammatory rheumatic diseases

## Abstract

There is an increased prevalence of atherosclerotic cardiovascular disease (ASCVD) in patients with inflammatory rheumatic diseases (IRD) including rheumatoid arthritis, systemic lupus erythematosus, psoriatic arthritis, and systemic sclerosis. The mechanism for the development of ASCVD in these conditions has been linked not only to a higher prevalence and undertreatment of traditional cardiovascular (CV) risk factors but importantly to chronic inflammation and a dysregulated immune system which contribute to impaired endothelial and microvascular function, factors that may contribute to accelerated atherosclerosis. Accurate ASCVD risk stratification and optimal risk management remain challenging in this population with many barriers that include lack of validated risk calculators, the remitting and relapsing nature of underlying disease, deleterious effect of medications used to manage rheumatic diseases, multimorbidity, decreased mobility due to joint pain, and lack of clarity about who bears the responsibility of performing CV risk assessment and management (rheumatologist vs. primary care provider vs. cardiologist). Despite recent advances in this field, there remain significant gaps in knowledge regarding the best diagnostic and management approach. The evolving field of Cardio-Rheumatology focuses on optimization of cardiovascular care and research in this patient population through collaboration and coordination of care between rheumatologists, cardiologists, radiologists, and primary care providers. This review aims to provide an overview of current state of knowledge about ASCVD risk stratification in patients with IRD, contributing factors including effect of medications, and review of the current recommendations for cardiovascular risk management in patients with inflammatory disease with a focus on hypertension as a key risk factor.

## Introduction

Cardiovascular diseases (CVD) are the leading cause of death globally.^1^ There is a higher prevalence of a broad range of CVD including atherosclerotic cardiovascular disease (ASCVD) in patients with inflammatory rheumatic diseases (IRD) compared to the general population.^2–4^

**Figure 1.**
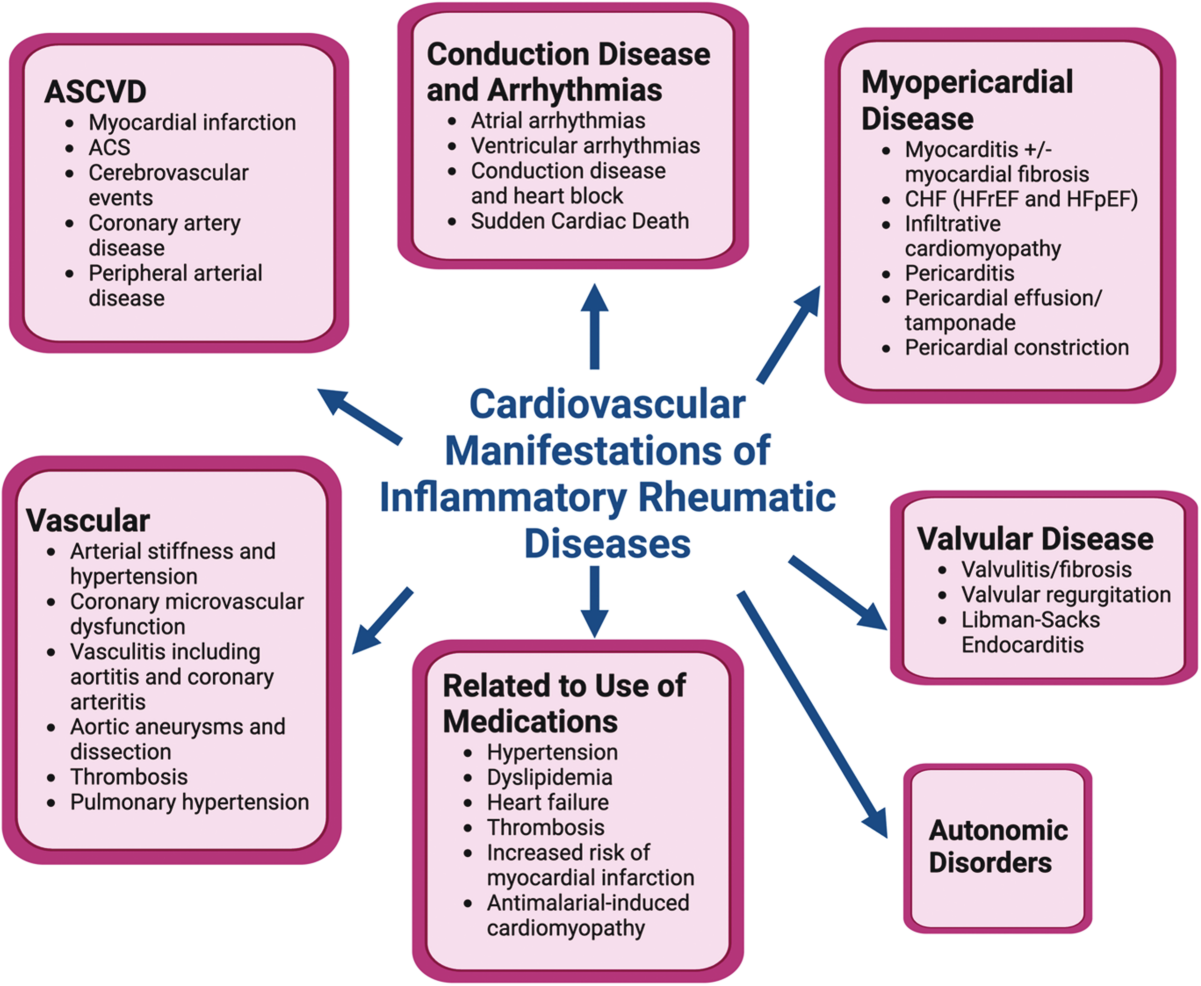
Range of cardiovascular problems in inflammatory rheumatic diseases. ASCVD, atherosclerotic cardiovascular disease; ACS, acute coronary syndromes; CHF, congestive heart failure; HfrEF, heart failure with reduced ejection fraction; HfpEF, heart failure with preserved ejection fraction.

The mechanism for development of ASCVD in the presence of systemic inflammatory disease has been linked not only to a higher prevalence and undertreatment of traditional cardiovascular (CV) risk factors but importantly to chronic inflammation and a dysregulated immune system which contribute to impaired endothelial and microvascular function, factors that may contribute to accelerated atherogenesis.^5–7^ Other than a predisposition to premature ASCVD which is the focus of this review, patients with IRD are at high risk for a wide range of other cardiac and vascular problems, including myocarditis, ischemic, and non-ischemic cardiomyopathy, pericardial disease, valvulitis, and valvular dysfunction, conduction disease and arrhythmias, pulmonary hypertension, and vasculitis. A hypercoagulable state may be present in some IRDs, predisposing patients to thrombotic events including vascular thrombosis and nonbacterial thrombotic endocarditis (Figure 1).

The increased prevalence of CVD in patients with IRDs and the worse associated CV outcomes, highlight CVD prevention and management as a vital component of care in this patient population. The evolving field of Cardio-Rheumatology focuses on the optimization of cardiovascular care and research in patients with IRDs through collaboration and coordination between rheumatologists, cardiologists, radiologists, and primary care providers. In this review, we aim to provide an overview of current state of knowledge about ASCVD risk stratification in patients with IRD, contributing factors including effect of medications, and review of the current recommendations for risk management in this population with a focus on hypertension (HTN) as a potent and modifiable risk factor.

## CARDIOVASCULAR RISK IN RHEUMATIC DISEASES

Increased risk of CVD morbidity and mortality has been reported in a wide range of IRDs. Rheumatoid arthritis (RA), the most common type of chronic inflammatory arthritis, affects approximately 1% of the adult population and has been consistently shown to be associated with an increase in CV risk. CVD mortality in RA has been reported to be increased by 50% compared to the general population.^8^ Patients with RA have also been reported to have a 1.7-fold increased risk of myocardial infarction (MI) compared to those without RA^2^ and poorer prognosis after an MI.^9^ A 2-fold increased risk for developing congestive heart failure (CHF), both due to HF with reduced ejection fraction (HFrEF) and with preserved ejection fraction (HFpEF),^10,11^ have also been reported in patients with RA compared to those without RA, a risk that is not fully explained by presence of ischemic heart disease and is associated with underlying disease activity and rheumatoid factor seropositivity.^11^ Furthermore, there is a higher prevalence of atrial fibrillation and strokes in individuals with RA, especially in those with elevated markers of disease activity including severe extra-articular RA-related manifestations, such as rheumatoid nodules (HR 3.29, 95% CI 1.98–5.48) and elevated inflammatory markers (HR 2.04, 95% CI 1.19–3.5).^12,13^ Systemic lupus erythematosus (SLE) is an autoimmune systemic condition, predominantly affecting women, with multiple organ involvement including the heart. In a recent nationwide Danish cohort study, patients with SLE had a higher associated risk of CHF, ASCVD, arrhythmias, and sudden cardiac death compared to matched control subjects.^4^ Psoriatic arthritis (PsA), a seronegative type of inflammatory arthritis, affects a third of patients with psoriasis. Both psoriasis and PsA are strongly associated with obesity and metabolic syndrome. A meta-analysis of observational studies in patients with PsA reported 43% and 22% increase in CV and cerebrovascular morbidity, respectively.^14^ Increased risk of CHF in PsA was associated with a combination of known traditional CV risk factors and measures of disease activity, particularly in non-ischemic heart failure, suggesting that the effect of inflammation on CHF may be partially independent of atherosclerotic disease.^15^ Increased risk is also observed in a wide range of other rheumatic inflammatory conditions, such as ankylosing spondylitis, systemic sclerosis, giant cell arteritis, and Takayasu arteritis, though less well studied.

## CHALLENGES OF MANAGING ATHEROSCLEROTIC CARDIOVASCULAR RISK IN RHEUMATIC DISEASES

**Figure 2.**
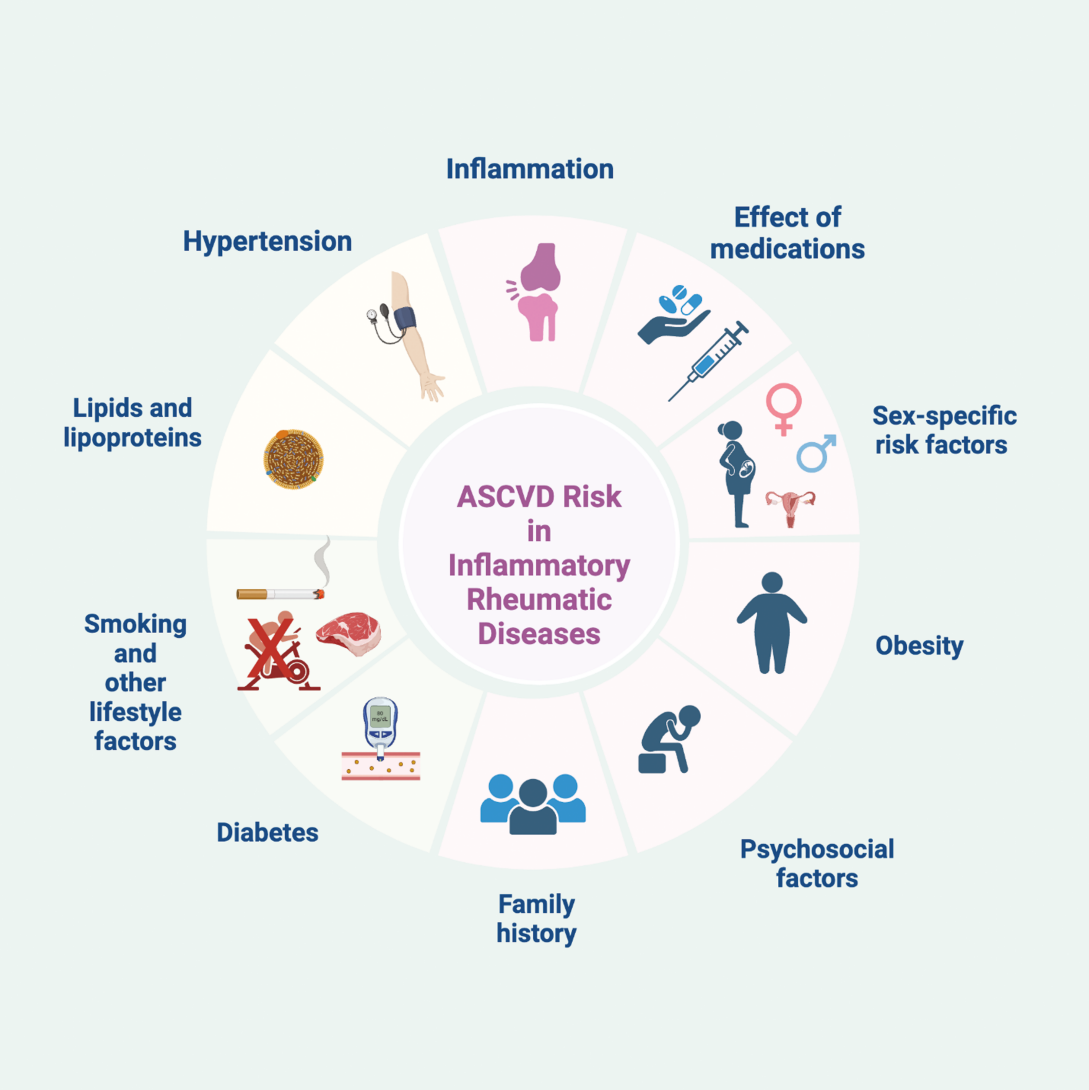
Risk factors for atherosclerotic cardiovascular disease in patients with inflammatory rheumatic diseases.

Despite growing knowledge of the increased ASCVD risk and its mechanisms over the last decade, there remain significant gaps in diagnosis and treatment of traditional CV risk factors including HTN in this population (Figure 2).^16,17^ CV risk assessment and management in patients with underlying IRDs can be challenging due to a number of different barriers including lack of validated risk calculators, the remitting and relapsing nature of underlying inflammatory disease, deleterious effect of medications used to manage rheumatic diseases, multimorbidity, decreased mobility due to joint pain, and lack of clarity about who bears the responsibility of performing CV risk assessment and management (rheumatologist vs. primary care provider vs. cardiologist).

## CURRENT STATE OF ASCVD RISK STRATIFICATION IN PATIENTS WITH RHEUMATIC DISEASE

The commonly used ASCVD risk calculators, such as the Framingham Risk Score and the Pooled Cohort Equations typically underestimate risk in this patient population.^16^ Efforts to include nontraditional risk factors, disease-specific parameters, multipliers, and biomarkers have not yet been successful at improving risk estimates for primary prevention.^18,19^ Exposure to traditional risk factors, such as HTN, can vary markedly over time in patients with a chronic relapsing-and-remitting disease partly due to variable disease activity and treatment-related factors, such as use of non-steroidal anti-inflammatory drugs (NSAIDs), glucocorticoids, and selected disease-modifying anti-rheumatic drugs (DMARDs) that can affect blood pressure such as leflunomide.^17^ Cumulative exposure overtime to risk factors, such as dyslipidemia and HTN, are better able to quantify ASCVD risk,^18^ though these dynamic risk factors are difficult to capture in a single point-in-time model, likely contributing to the poor performance of current snapshot risk calculators. Additionally, some risk factors behave paradoxically in the inflammatory milieu; for example, lower Body Mass Index (BMI) in RA has been associated with worse CV outcomes.^20^ Similarly, lipid levels may appear falsely low in the presence of active inflammation and start to rise once inflammation is better controlled, the so-called lipid paradox.^21^

The European League Against Rheumatism (EULAR) recommended a multiplier of 1.5 to the calculated score using traditional ASCVD risk models for RA.^22^ Despite this recommendation, validation studies have shown that using this multiplication factor does not significantly improve risk prediction.^23,24^ Literature to support recommendations for other rheumatic conditions is even more scarce. As such, EULAR did not endorse the use of any particular risk calculators or multipliers for SLE or other IRDs in their latest recommendations in 2021 due to lack of validated disease-specific tools. Instead, they recommended a thorough assessment of traditional and disease-specific factors to guide prevention efforts.^25^ The American College of Cardiology/American Heart Association (ACC/AHA) 2018 guidelines for ASCVD risk management do recognize chronic inflammatory conditions, such as RA, as a risk-enhancing factor to guide clinician–patient risk discussion in favor of use of statins in patients that would otherwise be placed in an intermediate risk category.^26^ Similarly, the Canadian Cardiovascular Society 2021 guidelines for the management of dyslipidemia, recognize individuals with inflammatory diseases to be at higher ASCVD risk and suggest screening for dyslipidemia regardless of age for all individuals with systemic inflammatory disease.^27^ Use of risk enhancers, such as elevation in lipoprotein (a) (Lp(a)), an LDL-like atherogenic lipid particle, and high-sensitivity C-Reactive Protein (hs-CRP), an inflammatory biomarker, are recommended to help further risk stratify patients in the general population. Data from both primary and secondary ASCVD prevention studies suggest that Lp(a)-associated ASCVD risk is enhanced in the presence of inflammation, highlighting the utility of these risk enhancers in patients with systemic inflammatory disease.^28,29^ In addition, vascular imaging biomarkers can also be used as risk modifiers to further fine-tune ASCVD risk assessment. There is ample evidence in the general population for the use of noninvasive vascular imaging for detection of subclinical atheroma, such as non-contrast coronary artery calcium measurement and/or carotid ultrasound, to improve appropriate selection of patients that are at high risk of ASCVD events.^30–35^ In the absence of a reliable clinical risk calculator in patients with IRD, consideration of additional risk enhancers, such as Lp(a), along with more liberal use of vascular imaging to screen for subclinical atherosclerosis appears to be the most effective way of risk stratification in patients that would otherwise be considered at low or intermediate risk by traditional risk calculators.

## INTERSECTION WITH SEX AND GENDER

It is important to recognize that many IRDs, including RA and SLE, disproportionately affect women. Of the approximately 8% of the general population affected by IRDs, 80% are women,^36^ with many of them affected by these conditions starting at a young age, exposed to chronic inflammation, effect of medications, as well as debilitating physical symptoms and psychological burden of a disrupted sense of identity, loss of independence, societal stigma, and complicated reproductive health issues that may coexist. Traditional ASCVD risk calculators, blind to these important factors, would commonly place them in a low-risk category, leading to underestimation and undertreatment of their true ASCVD risk. This underscores the need for a more thorough ASCVD risk assessment in this population than offered by traditional risk calculators.

## EFFECT OF MEDICATIONS USED IN IRD ON CV RISK

Medications used in the treatment of IRDs have the potential to further alter ASCVD risk. DMARDs are the cornerstone of treatment of inflammatory arthritis, aiming to control systemic inflammation leading to improvement in symptoms and prevention of joint and extra-articular damage. Corticosteroids and NSAIDs are typically used either as bridging therapy for short-term effects or together with DMARDs for control of symptoms and disease activity. Over the past two decades, a variety of targeted biologic and synthetic DMARD therapies have been approved for the management of rheumatic conditions. Despite effective control of inflammation, research has shown that different therapies have differential effects on CV risk. While medications such as systemic corticosteroids, NSAIDs, and some DMARDs, like leflunomide, may contribute to an increased CV risk, others, like methotrexate and anti-Tumour Necrosis Factor (TNF) therapies, have been shown to have cardioprotective effects.^37–39^ Corticosteroid use is associated with a variety of adverse CV effects including HTN, premature atherosclerosis, risk of MI, arrhythmias, and HF.^8^ However, observational studies in RA suggested that low-dose prednisone (<5 mg daily) used as maintenance therapy does not increase CV risk.^40,41^ Use of both nonselective and cyclo-oxygenase-2 (COX-2)-selective NSAIDs have been found to increase the risk of CV events. A number of studies, including a large nationwide Danish cohort study, have found that the CV risk associated with NSAID use in RA patients was significantly lower than in non-RA patients, possibly related to potential inflammatory mechanisms shared between RA and CV disease or better pain control and subsequent increase in mobility.^42^ Tofacitinib, a targeted synthetic DMARD that inhibits Janus Kinase (JAK), has been linked to higher risk of major adverse cardiovascular events (MACE) compared to anti-TNF therapy among patients with RA with a higher CV risk. In the ORAL surveillance study, a safety, non-inferiority trial mandated by the Food and Drug Administration (FDA), patients with RA aged ≥50 years who had at least 1 CV risk factor were randomized to tofacitinib or to a TNF inhibitor. The study showed that the risk of developing MACE and venous thromboembolism was higher in those on tofacitinib than TNF inhibitors, thus failing its non-inferiority criteria.^43^ This study led to the addition of a label warning by regulatory agencies, including Health Canada and the FDA, assigned to all JAK inhibitors for all clinical indications. A number of anti-inflammatory medications including canakinumab (anti-IL 1b) have been shown in randomized controlled trials to be effective in secondary prevention of ASCVD events in the general population^44,45^ but efficacy and safety with regards to CVD outcomes in IRD have not been studied. Finally, medications such as the antimalarial drugs, chloroquine and hydroxychloroquine, can have both important cardioprotective as well as uncommon, but potentially life-threatening cardiotoxic side effects in a small number of patients who develop cardiomyopathy, typically with long-term use.^46^

## A FOCUS ON HYPERTENSION IN PATIENTS WITH IRD

HTN is a leading modifiable risk factor for CV death. Nearly half of adults in the US (48.1%, 119.9 million) have HTN, defined by the ACC/AHA guidelines as a systolic BP > 130 mmHg or a diastolic BP > 80 mmHg, or are taking medication for HTN.^47,48^ HTN control is a key component of ASCVD risk reduction and significantly lowers risk both in primary as well as in secondary prevention and reduces mortality from CV events.^49,50^

Numerous studies have reported a higher prevalence of HTN in patients with IRD than in the general population.^51–55^ The exact prevalence is difficult to determine with wide ranges reported in various studies, partially related to underdiagnosis of HTN in this population, impact of disease-specific therapies, varied sample size and significant differences in definition of HTN used over time and across jurisdictions due to variations in expert society guidelines and criteria. In patients with RA, studies have shown conflicting results with some case-control studies showing a significant increase in prevalence of HTN compared to healthy individuals,^51,56^ whereas other studies have not.^57,58^ The best evidence for higher prevalence of HTN comes from large community-based studies demonstrating that HTN was significantly more prevalent in patients with RA than the general population.^51,59^ One study found an increased prevalence of HTN in patients with RA (31%) compared to the general population (17%).^51^ In another study, prevalence of HTN in patients with RA was found to be 70% in both men and women of all age groups with a significant proportion (35% of males and 41% of females) undiagnosed and untreated, higher than that of the general population. Age, elevated BMI, and steroid use were independently associated with presence of HTN.^55^ Importantly, undiagnosed HTN was much more common in younger patients with RA.^55^ Similarly, in PsA and ankylosing spondylitis, large population-based case-control studies have demonstrated a higher prevalence of HTN compared to the general population.^60–62^ In SLE, HTN has been found to be more prevalent than in healthy individuals, particularly in younger women.^54,63^ HTN has been shown to be prevalent in patients with scleroderma, especially with renal involvement.^64^ Additionally, studies have shown that BP fluctuates considerably in patients with IRD due to variations in disease activity and subsequent change in medications including increased use of NSAIDs and corticosteroids, as well as potential kidney involvement in some IRDs at various stages of disease.^65^

Despite its high prevalence, HTN is underrecognized and undertreated among patients with IRD.^16,53^ Many patients are not prescribed appropriate antihypertensive therapy and many of those taking antihypertensive medications are not at the recommended treatment targets. In their study of frequencies of indication for CV preventative drugs in patients with inflammatory joint disease, Ikdahl *et al.* found an indication for antihypertensive treatment in 53% of patients of whom 48% were untreated. They also found that of those treated with antihypertensive medications, approximately 50% were not at guideline-recommended treatment targets recommended for the general population.^66^

There are multiple potential reasons for the underdiagnosis and undertreatment of HTN and other traditional CV risk factors in this patient population. Studies that have evaluated barriers to CV risk management among rheumatologists and PCPs have identified lack of clarity about who is responsible for CV risk management (rheumatologist or PCP), lack of time, lack of knowledge of current guidelines for CV risk management, and lack of care coordination as some of the main perceived reasons.^67,68^ Additionally, some clinicians may not be aware of the increased risk in patients with IRD. Due to frequent visits to their rheumatologist centered on joint and extra-articular symptoms, patients may not visit their PCP regularly and may be less regularly screened for various common risk factors. Finally, polypharmacy and “medication-fatigue” in patients with underlying chronic inflammatory disease can also contribute to this problem.

## MECHANISMS OF HYPERTENSION IN IRD

**Figure 3.**
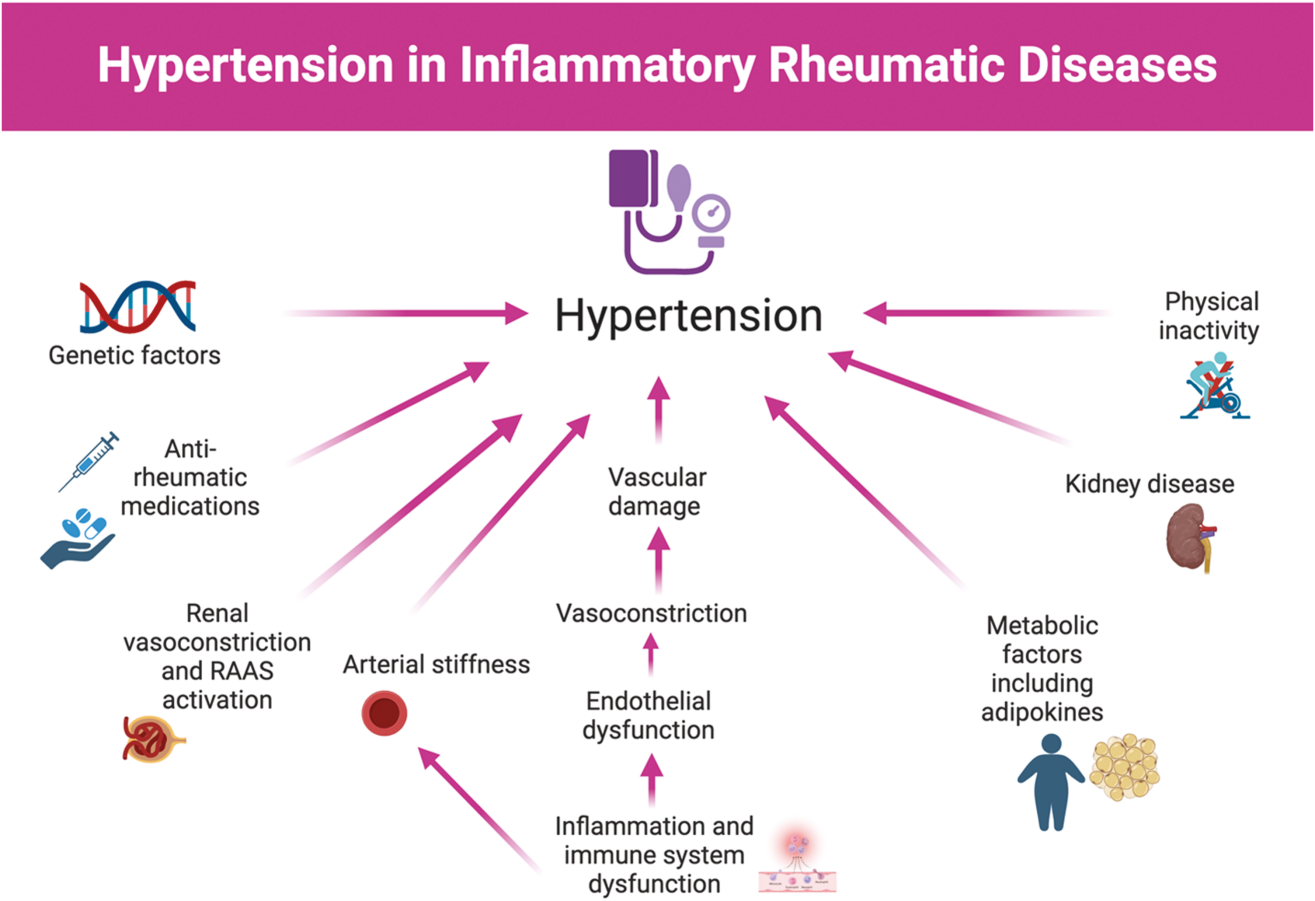
Factors contributing to the development of hypertension in inflammatory rheumatic diseases; RAAS, renin–angiotensin–aldosterone system.

The mechanisms that contribute to HTN in patients with IRDs are multifactorial and are not fully understood but are at least in part dependent on disease-related factors including degree of inflammation, renal involvement, and disease-specific therapeutics (Figure 3). A number of overlapping genetic and environmental risk factors exist for development of both IRD and HTN.^69^ For example, adipokines such as leptin, secreted by white adipose tissue, have been implicated to have proinflammatory effects in autoimmune conditions such as SLE and RA.^70^ Leptin is also known to increase BP through impaired natriuresis, vascular endothelial dysfunction, oxidative stress, and increased renal tubular sodium absorption.^71^ Inflammation and immune-mediated damage of the vessel wall can lead to endothelial dysfunction and arterial stiffness leading to HTN.^72^ In conditions such as SLE, renal disease and progressive kidney dysfunction may also contribute to increased risk of HTN. Lupus nephritis occurs in nearly 50% of patients with SLE and has a strong correlation with HTN, though HTN may develop independently of lupus nephritis.^73^ Additionally, renal vasoconstriction and ischemia-induced activation of the renin–angiotensin–aldosterone system (RAAS) are important factors in development of HTN in vasculitis affecting renal arteries and in scleroderma renal crisis.

Physical inactivity exacerbated by joint pain, stiffness, and permanent joint damage may lead to a sedentary lifestyle and obesity, synergistically contributing to HTN in these patients. Recommendations for even mild physical exercise including walking or swimming that may be better tolerated in patients with joint disease should be encouraged. Design and implementation of specific structured exercise programs for these patients may provide more pronounced benefits.

## IMPACT OF MEDICATIONS USED IN IRD ON HYPERTENSION

The effect of medications used for the treatment of underlying IRD must also be considered. All NSAIDs, both nonselective and selective, at doses required to control inflammation, can increase blood pressure though the effect on BP is variable.^74–79^ NSAIDs can also diminish the efficacy of many antihypertensive medications, with the exception of calcium channel blockers (CCB).^74,75^ There has been an ongoing interest in identifying which NSAIDs have the best safety profile with regard to HTN and CV outcomes, but this remains uncertain. A meta-analysis of randomized trials studying the effect of NSAIDs on blood pressure found that when pooled, NSAIDs elevated mean blood pressure by 5.0 mmHg (95% CI, 1.2–8.7 mmHg).^76^ Additionally, long-term NSAID use may also have a negative impact on kidney function, further contributing to HTN. Using the smallest dose required for the shortest duration of time possible with careful monitoring of BP is therefore recommended with all NSAIDs. There are no RCTs evaluating the effect of corticosteroids on BP in patients with IRD. Long-term (>6 months) and moderate-to-high doses (prednisone > 7.5 mg daily) have been associated with increased risk of developing HTN in RA, independently of other risk factors.^80^ The DMARD leflunomide has been reported to induce HTN in 2–5% of patients with RA, thought to be related to an increase in autonomic sympathetic tone, displacement of free fraction of any concomitant NSAID from protein binding, and increasing salt and water retention.^81^ Cyclosporine can also induce HTN through vasoconstrictive effect on the renal circulation resulting in reduced kidney function and should be avoided if possible in patients with HTN.^82^ The effect of biologic therapies on BP remains uncertain due to paucity of data, though TNF inhibitors have been shown to have beneficial effects on arterial stiffness in RA.^83^ In a small study of patients with RA, treatment with the TNF inhibitor infliximab was shown to result in a significant reduction in BP which was also associated with reduction in disease activity,^84^ although the study was limited by short duration of follow up.

## BP TARGETS IN IRD

Despite known high prevalence of HTN and associated increased ASCVD risk, there is little data on optimal threshold for initiation and BP targets of therapy for patients with IRD and typically recommendations for the general population are applied to this group of patients. The 2023 European Society of Hypertension (ESH) guidelines recognize immune-mediated inflammatory diseases as being associated with increased prevalence of HTN that is underdiagnosed and poorly controlled, but no specific recommendations with regard to specific systolic and diastolic BP criteria for diagnosis, thresholds for initiation of therapy or for targets of therapy were suggested for this population. The 2017 ACC/AHA guidelines defined HTN at ≥ 130/80 mmHg, however, no special consideration or recommendation was provided for management of HTN in patients with IRDs. Tselios *et al.* studied the impact of this new definition of HTN on patients with SLE and showed that patients with a sustained mean BP of 130–139/80–89 mmHg over 2 years had a 2.5-fold increased risk of ASCVD events compared with those with BP < 130/80 mmHg. After adjustment for traditional and disease-related atherosclerotic risk factors, this level of BP conferred a 73% increased risk for atherosclerotic vascular events and therefore, they proposed a treatment BP target < 130/80 mmHg in patients with SLE to effectively reduce CV risk.^85^ The EULAR is also now recommending a target BP < 130/80 mmHg in patients with SLE. Due to paucity of data and in keeping with the ESH guidelines, the EULAR recommendation for management of HTN in other IRDs including RA, PsA, and ankylosing spondylitis is to follow recommendations used for the general population.^22,25^ BP thresholds for initiation of antihypertensive therapy and treatment targets, even in the general population, remain to be harmonized with variations across different practice guidelines.^48,86^ More research, particularly in the IRD population, is needed to define the best initiation threshold and targets of therapy.

## CHOICE OF ANTIHYPERTENSIVE DRUGS IN IRD

The optimal choice of antihypertensive medication in patients with IRD is not well studied. EULAR recommends antihypertensive management in accordance with national guidelines for the general population. The 2023 ESH guidelines recommend lowering BP to targets recommended in the general population, preferentially with CCB and RAAS inhibitors because of the evidence of an overactive RAAS in these conditions,^87^ while also focusing on reducing systemic inflammation, and avoiding high dose NSAIDs. Multiple studies have demonstrated that concomitant use of nonselective NSAIDs attenuates the antihypertensive effect of angiotensin-converting enzyme inhibitors, angiotensin receptor blockers, diuretics, and beta blockers.^76,88,89^ However, NSAIDs do not appear to alter the antihypertensive effect of CCBs^90,91^ and, therefore, in patients with HTN who require daily NSAID use, a dihydropyridine CCB such as amlodipine should be considered. Beta blockers may trigger or worsen psoriasis^92^ and should be used with caution if there is a compelling indication such as CHF. Similarly, use of beta blockers is avoided in patients with Raynaud’s as this can worsen symptoms and CCBs are favored due to their beneficial effects on Raynaud’s. Future research is needed to fill the knowledge gaps in this area and help delineate the most effective regimens in various IRDs with regard to BP control and long-term CV outcomes.

## PRACTICAL IMPLICATIONS FOR SCREENING, DIAGNOSIS, AND TREATMENT OF HTN IN IRD

Given the high prevalence of HTN and its underdiagnosis and undertreatment in patients with IRD, it is prudent that patients undergo regular annual screening with their PCP and at visits with their rheumatologist, particularly after change in medications. Careful reevaluation of blood pressure and its control should also be considered at the time of development of comorbidities such as ASCVD, diabetes, or proteinuria. It is also advisable that for patients who are physically and financially able to do home BP measurements, to have ongoing self-monitoring to help exclude both white coat and masked hypertension. When unclear, use of Ambulatory Blood Pressure Monitoring can be helpful in more precise BP assessment, though access particularly related to cost coverage may be limiting in some jurisdictions. With regard to the choice of antihypertensive therapy, following societal guidelines with consideration of comorbidities, potential contraindications related to other conditions, and interactions with concurrent medications should be considered. As for targets of therapy, national societal guidelines recommendations are to be followed for the general population with the exception of a BP target of <130/80 mmHg for SLE given specific EULAR recommendations in this condition.^25^

## CONCLUSIONS AND FUTURE DIRECTIONS

Individuals with IRD are at higher risk of developing ASCVD and experiencing worse CV outcomes than the general population. Despite significant advances over the last decade in understanding pathophysiological mechanisms underlying this increased risk, accurate ASCVD risk stratification and management remains suboptimal. Key overarching strategies for prevention of CV complications include aggressive management of traditional risk factors as well as achievement of effective control of systemic inflammation. Despite HTN being one of the most important and prevalent modifiable traditional risk factors in patients with IRD, it remains underdiagnosed, undertreated, and under-researched in this population. There is increasing recognition that an interdisciplinary model of care is crucial for optimal management of CV risk in these patients. Fostering collaborations between rheumatologists and cardiologists, such as that done in dedicated Cardio-Rheumatology programs, provides a valuable space for designing tailored screening protocols and management strategies for individuals with IRD, while allowing for combined research efforts to fill current knowledge gaps and improve quality of cardiovascular care offered to this patient group.

## Data Availability

No new data were generated or analysed in support of this research.
